# Supplementary material for: Laboratory Cross-Sensitivity Evaluation of Low-Cost Electrochemical Formaldehyde Sensors
Source: Sensors (Basel). 2025 May 14;25(10):3096. doi: 10.3390/s25103096 (PMC12115294; doi:10.3390/s25103096)
Supplement: Supplementary file 1 [file sensors-25-03096-s001.zip › sensors-3616248-supplementary.pdf]

Laboratory cross-sensitivity evaluation of low-cost electrochemical formaldehyde sensors

Supplementary Materials

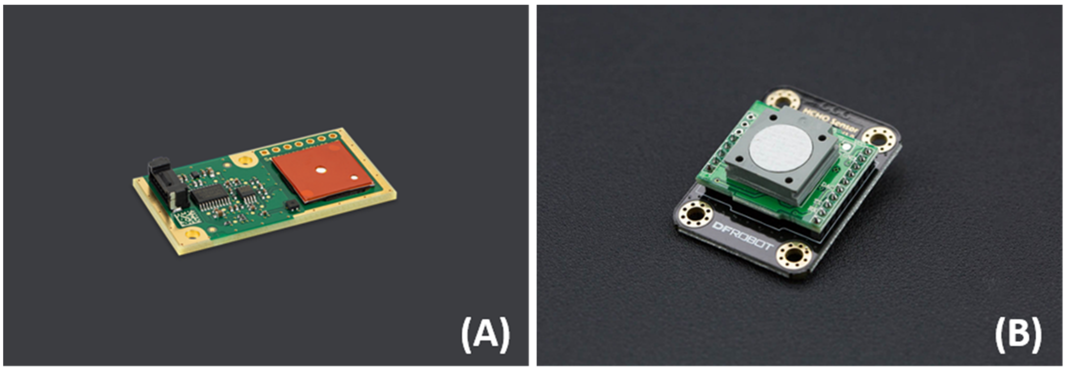

Figure S1. Electrochemical sensors evaluated in the study: (A) Sensirion SFA30; (B) DFRobot Gravity.

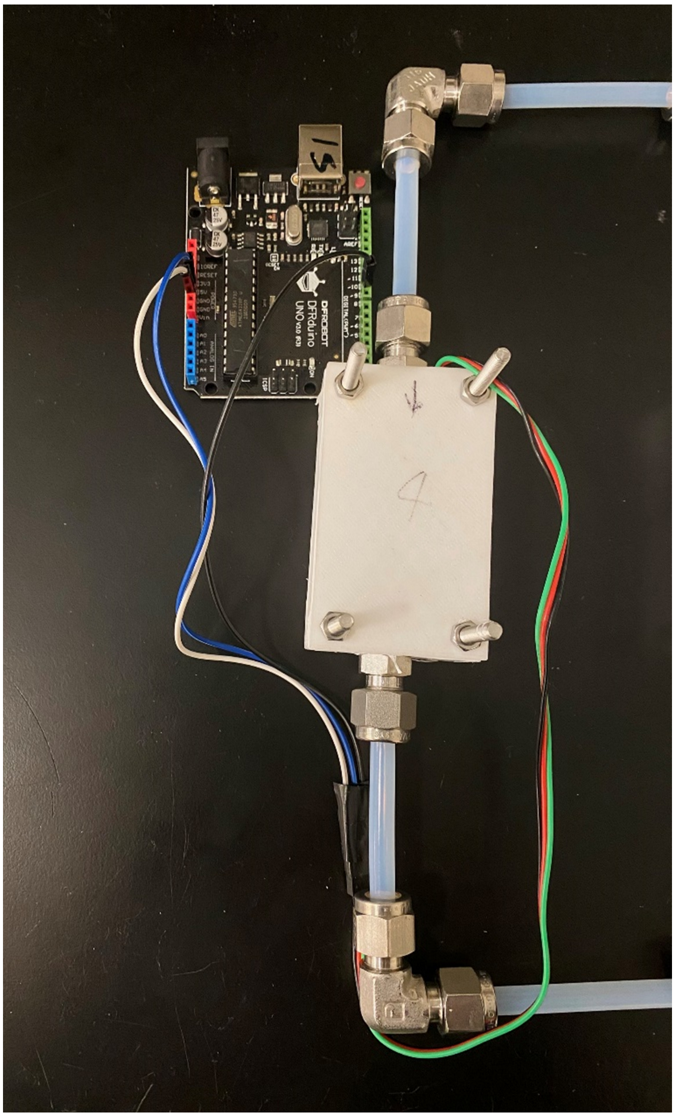

Figure S2. 3D-printed PLA chamber for sensor evaluation.

Table S1. Validation of the passive samplers in the anatomy lab

Target gas		Formaldehyde	Methanol	IPA
Sampler name		Radiello®	Assay Technology® 545 Methanol Badges	Assay Technology® 566 Organic Vapor Monitors
Analysis method		High-performance liquid chromatography	Gas chromatography flame ionization detector	
Method LOD		0.407 ppb (4.5 days)	1.1 ppm (8h)	0.072 ppm (8h)
Validation #1	Sampler concentration *	262 ppb	< 2.8 ppm	0.77 ppm
	Sampling time	180 min		
Validation #2	Sampler concentration *	343 ppb	< 1.9 ppm	0.73 ppm
	Sampling time	264 min		
Validation #3	Sampler concentration *	216 ppb	< 1.8 ppm	0.49 ppm
	Sampling time	276 min		

* All sampler concentrations were obtained by subtracting the sampler blank measurement from each measurement. The sampler blank measurement was obtained by placing one sampler in the bag inside the refrigerator.

Table S2. Sensor responses in the repeated concentration-only tests

**BBCEAS**	**SFA30 S1**	**SFA30 S2**	**SFA30 S3**	**SFA30 S4**	**BBCEAS**	**Gravity S1**	**Gravity S2**
25.1±2.08	125±1.25	109±0.929	130±1.01	111±1.10	13.5±2.09	14.0±0.00	15.0±0.00
37.1±2.28	192±1.25	159±1.16	199±1.29	174±1.25	27.2±2.06	15.0±0.00	19.0±0.00
49.3±2.92	256±1.30	212±1.33	269±1.54	234±1.15	41.7±1.23	20.5±0.500	24.0±0.00
60.8±2.97	326±1.64	265±1.19	346±1.82	301±1.55	56.1±2.52	25.0±0.102	29.0±0.00
24.2±2.80	120±1.12	107±0.890	124±1.16	108±1.00	11.3±2.10	14.0±0.00	15.0±0.00
35.1±2.10	190±1.13	158±1.01	194±1.16	173±1.08	25.3±2.29	15.0±0.00	19.0±0.00
47.5±1.81	261±1.41	215±1.14	272±1.37	240±1.31	40.6±1.95	20.0±0.143	24.0±0.00
60.0±2.25	334±1.42	271±1.21	352±1.41	309±1.34	53.9±1.70	24.7±0.450	29.0±0.00
23.2±1.72	113±1.07	90.0±0.926	108±0.986	95.0±0.931	12.2±1.76	14.0±0.00	15.0±0.00
35.9±2.19	178±1.49	141±1.07	175±1.21	157±1.29	26.7±2.57	15.0±0.00	19.0±0.144
48.9±2.13	247±1.36	198±1.12	251±1.23	222±1.17	42.7±2.35	20.5±0.500	24.0±0.00
59.6±1.66	317±1.25	252±1.16	329±1.57	290±1.36	57.6±1.92	25.0±0.143	29.0±0.00
86.9±2.01	896±2.04	772±5.18	1031±4.20	888±3.31	80.9±2.03	40.9±0.260	49.0±0.00
130±2.07	1540±4.58	1241±8.72	1751±9.24	1507±8.31	122±1.73	59.0±0.143	69.1±0.331
173±0.982	2209±6.77	1701±11.4	2485±14.4	2130±10.9	161±1.26	77.9±0.319	94.0±0.102
255±1.37	3730±15.5	2762±20.2	4169±23.3	3560±20.0	249±1.74	137±0.755	156±0.496
113±1.39	976±4.74	816±6.06	1102±4.54	952±4.67	98.0±2.17	43.0±0.00	19.0±0.00
168±1.24	1629±6.72	1294±9.36	1844±10.2	1591±8.52	152±2.07	62.0±0.222	69.3±0.455
223±1.39	2682±16.5	1855±20.3	2675±33.6	2649±35.4	203±2.51	79.0±0.102	94.8±0.427
326±2.05	4761±14.8	2862±17.3	4360±20.8	4531±20.0	320±1.99	141±0.580	161±0.455
109±1.76	888±1.97	738±4.16	1012±3.87	869±2.88	99.6±1.69	43.3±0.434	47.5±0.130
166±2.24	1622±5.89	1266±8.55	1799±9.91	1585±9.05	152±1.84	62.4±0.273	67.5±0.386
220±1.53	2307±8.44	1736±9.39	2557±12.4	2211±12.3	206±2.16	83.2±0.452	91.6±0.440
319±1.95	3967±11.0	2882±16.1	4376±18.8	3747±16.7	319±1.98	147±0.357	155±0.319

Note: This table shows the formaldehyde measurements from the BBCEAS and the sensors (average ± standard deviation). All units are ppb.

Table S3. Individual sensor performance metrics in the concentration-only tests

**Metrics**	**SFA30 S1**	**SFA30 S2**	**SFA30 S3**	**SFA30 S4**	**Gravity S1**	**Gravity S2**
Slope	14.4±0.536	9.82±0.339	14.9±0.538	13.8±0.497	0.438±0.0162	0.486±0.0196
Intercept	-430±79.6	-223±50.4	-409±80.0	-413±73.9	2.93±2.30	4.78±2.77
R^2	0.970	0.974	0.972	0.972	0.971	0.965
RMSE (ppb)	16.1	14.9	15.6	15.6	16.1	17.5
NRMSE (%)	5.32	4.93	5.14	5.15	5.20	5.65
LOD (ppb)	4.58	6.58	5.93	5.52	14.5	4.73
CV (%)	4.73	3.99	3.83	5.46	1.55	0.714

Note: This table shows metrics ± the standard deviation of the three repeated tests for each concentration.

Table S4. Individual sensor cross sensitivity to outdoor trace gases and the BBCEAS variations

	**CO**	**NO**	**NO_2**	**O_3**	**Isobutylene**
SFA30 S1	2.00±2.48	-33.7±4.84	-96.7±1.98	-99.8±0.0200	-0.240±3.01
SFA30 S2	0.609±2.25	-37.9±4.18	-98.3±0.413	-99.7±0.0300	-2.13±2.23
SFA30 S3	0.478±2.15	-36.2±4.43	-98.8±0.217	-99.8±0.0200	-0.860±2.24
SFA30 S4	0.894±2.13	-38.0±3.96	-98.7±0.205	-99.7±0.0300	-0.720±2.31
Gravity S1	17.7±4.27	0.590±3.99	3.09±3.36	5.73±5.40	6.30±5.93
Gravity S2	-8.96±3.30	-0.340±1.76	-1.82±3.46	5.43±6.14	-1.91±3.95
SFA30 average	1.00	-36.4	-98.1	-99.7	-0.988
Gravity average	4.39	0.125	0.635	5.58	2.20
BBCEAS variation for SFA30	7.41	8.49	9.17	9.72	8.09
BBCEAS variation for Gravity	6.77	10.1	7.99	10.7	7.16
BBCEAS formaldehyde concentration (ppb)	22.4-27.2	21.7-24.5	20.4-24.1	17.1-21.1	23.1-26.4

Note: All units are % unless noted. All tests were performed at 23.2±0.349 ℃ and 24.3±0.489% RH. This table shows the results ± standard deviation, based on three repeated tests for each gas (each test includes15-min of continuous stable sensor signal).

Table S5. Individual sensor cross sensitivity to indoor VOCs and variations in the BBCEAS-estimated concentration during these tests.

	**1 ppm methanol**	**2 ppm methanol**	**3 ppm methanol**	**0.5 ppm IPA**	**1 ppm IPA**	**1.5 ppm IPA**
SFA30 S1	3.86±0.166	0.0391±0.109	2.28±0.189	0.329±0.211	3.34±0.177	0.770±0.103
SFA30 S2	-2.74±0.360	-1.06±0.138	2.80±0.151	-0.143±0.367	3.34±0.151	0.480±0.0981
SFA30 S3	-2.92±0.239	-1.07±0.149	-0.527±0.410	0.0871±0.222	4.25±0.121	0.368±0.0984
SFA30 S4	-1.63±0.271	-0.788±0.111	3.97±0.193	-0.182±0.276	4.56±0.109	0.0428±0.132
Gravity S1	161±2.90	353±5.33	670±14.4	19.0±2.97	54.6±0.906	79.4±1.35
Gravity S2	118±3.55	238±8.25	337±15.5	18.8±2.73	53.1±1.06	77.2±0.415
SFA30 average	-0.855	-0.720	2.13	0.0228	3.89	0.415
Gravity average	140	295	503	18.9	53.8	78.3
BBCEAS variation for SFA30	0.794	0.490	0.604	0.614	0.576	0.684
BBCEAS variation for Gravity	0.417	0.516	0.675	0.503	0.521	0.490
BBCEAS formaldehyde concentration (ppb)	253-254	253-256	249-257	252-256	257-263	252-255

Note: All units are % unless noted. All tests were performed at 23.2±0.349 °C and 24.3±0.489% RH. This table shows the results ± standard deviation, based on three repeated tests for each gas (each test includes15-min of continuous stable sensor signal).
